# Un cas marocain d’érythrophagocytose blastique et LAL T de novo sans anomalie cytogénétique

**DOI:** 10.11604/pamj.2020.36.202.24477

**Published:** 2020-07-22

**Authors:** Sophia Kahouli, Hafid Zahid, Mohamed El khorassani, Saâd El kabbaj, Majid Benkirane, Nezha Messaoudi

**Affiliations:** 1 Laboratoire de Recherche et d´Analyses Médicales de la Fraternelle de la Gendarmerie Royale, Faculté de Médecine et de Pharmacie, Université Mohammed V, Rabat, Maroc,; 2 Service d´Hématologie-immunohématologie de l´Hôpital Militaire d´Instruction Mohammed V, Faculté de Médecine et de Pharmacie, Université Mohammed V, Rabat, Maroc,; 3 Service d'Hématologie et d'Oncologie Pédiatrique de l'Hôpital d´Enfants, Faculté de Médecine et de Pharmacie, Université Mohammed V, Rabat, Maroc,; 4 Pôle des Laboratoires de l´Hôpital Militaire d´Instruction Mohammed V, Faculté de Médecine et de Pharmacie, Université Mohammed V, Rabat, Maroc

**Keywords:** Erythrophagocytose blastique, leucémie aiguë lymphoblastique T, caryotype normal, absence d’anomalies cytogénétiques, hypertrophie gingivale, blastes activés, Erythrophagocytosis by blast cells, acute T-lymphoblastic leukemia, normal karyotype, absence of cytogenetic abnormalities, gingival hypertrophy, activated blast cells

## Abstract

L´érythrophagocytose blastique correspond à une hyperactivation des blastes. L´érythrophagocytose est retrouvée dans les hémopathies myéloïdes surtout avec la t (8;16). Dans ce travail nous présentons un cas exceptionnel d´érythrophagocytose blastique au cours d´une leucémie aigue lymphoblastique T sans anomalies cytogénétiques. A.Z âgée de 19 ans, l´examen à l´admission a trouvé un syndrome fébrile avec des vertiges et phosphènes, un syndrome tumoral avec une hypertrophie amygdalienne et gingivale. L´hémogramme a objectivé une hyperleucocytose (399,5 G/L), avec une anémie arégénérative (Hb: 9,3 g/dl) et thrombopénie (plaquettes: 40 G/L). Le myélogramme a montré 90% des blastes (MPO négative) avec des images d´érythrophagocytose blastique. L´immunophénotypage a confirmé une LAL T. L´analyse cytogénétique était normale. L´érythrophagocytose blastique dans une LAL T semblerait être une entité distincte nécessite la précision de l´impact de ces images sur le diagnostic, le pronostic voir même le traitement des LAL T.

## Abstract (en)

Erythrophagocytosis by blast cells is due to hyperactivation of blast cells. Erythrophagocytosis is associated with T cell myeloid hemopathies (8;16). This study shows an exceptional case of erythrophagocytosis by blast cells in a patient with acute T-lymphoblastic leukemia without cytogenetic abnormalities. We here report the case of A.Z, aged 19 years presenting with febrile syndrome with dizziness and phosphenes, tumor syndrome with amygdala and gingival hypertrophy. Blood count revealed hyperleukocytosis (399.5 G/L), with aregenerative anemia (Hb: 9.3 g/dl) and thrombocytopenia (platelet count: 40 g/L). Myelogram showed 90% of blast cells (MPO-negative) with erythrophagocytosis by blast cells images. Immunophenotyping confirmed T-cell LAL. Cytogenetic analysis was normal. Erythrophagocytosis by blast cells in patients with T-cell LAL appears to be a separate entity, hence the importance of images on diagnosis, prognosis and treatment of T-cell LAL.

## Introduction

L´érythrophagocytose blastique est traduit par une infiltration médullaire de blastes sous forme activée. La phagocytose peut intéresser les globules rouges ou les érythroblastes. Selon la littérature, l'érythrophagocytose par des blastes leucémiques est extrêmement rare. Elle est rapportée dans seulement moins de 1% des cas et est observée principalement dans les hémopathies myéloïdes. La cytologie est le seul test qui permet d’objectiver leurs présences [1, 2]. Nous présentons ici et à travers cette observation le premier cas marocain illustrant une association particulière de leucémie aigue lymphoblastique T de novo et une érythrophagocytose blastique sans anomalie cytogénétique.

## Patient et observation

A.Z est une patiente de 19 ans, originaire de Zagora-Maroc, c´est la 6^e^d´une fratrie de 8 filles. A.Z a été hospitalisée dans notre hôpital pour une altération de l´état général avec des signes neurosensoriels (vertiges et phosphènes). L´examen à l´admission a trouvé une fièvre à 38°C avec un syndrome tumoral fait: d´adénopathies axillaires, une hépatosplénomégalie et une hypertrophie amygdalienne et gingivale. L´hémogramme a révélé une anémie normochrome normocytaire arégénérative (l´Hb= 9,3 g/dl, réticulocyte= 60x10^9^/L) associée à une hyperleucocytose (leucocytes= 399 500/mm^3^), et une thrombopénie (plaquettes=40 000/mm^3^). Le bilan d´hémostase a été sans particularité. Le bilan biochimique a montré un syndrome de lyse spontané: acide urique=150 mg/l, LDH= 1716 UI/L (6 fois la normale) et une CRP à 8 mg/l. La fonction rénale (créatininémie à 380 µmol/l) a été normalisée après hydratation, la fonction hépatique n´a pas été perturbée. Les sérologies virales ont été négatives (VIH, VHC, VHB, TPHA-VDRL). La radiographie pulmonaire a été normale. Le frottis sanguin coloré au May Grünwald Giemsa (MGG) a retrouvé 93% de blastes avec un taux d´érythroblastes circulants de 3%. Les blastes ont été de taille inégale (petite, moyenne, grande) avec rapport nucléo-cytoplasmique élevé et noyau de contours souvent irréguliers, à chromatine fine et nucléolée. Le cytoplasme a été réduit, basophile et agranulaire contenant parfois quelques vacuoles. La réaction à la MPO (myéloperoxydase) a été négative.

### Diagnostic

**Figure 1:**
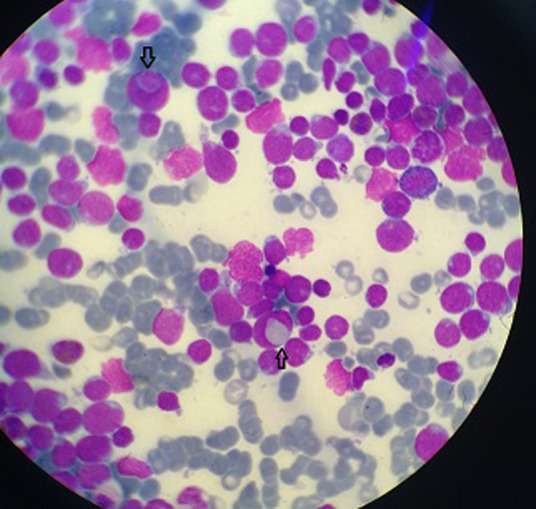
image montrant la phagocytose d’un un globule rouge et d’un érythroblaste par des lymphoblastes T (MGG× 100)

**Figure 2:**
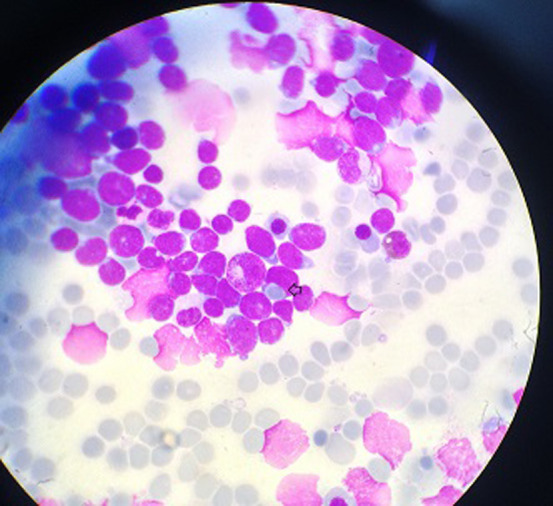
image montrant la phagocytose d’un un globule rouge par un lymphoblaste T (MGG× 100)

**Figure 3:**
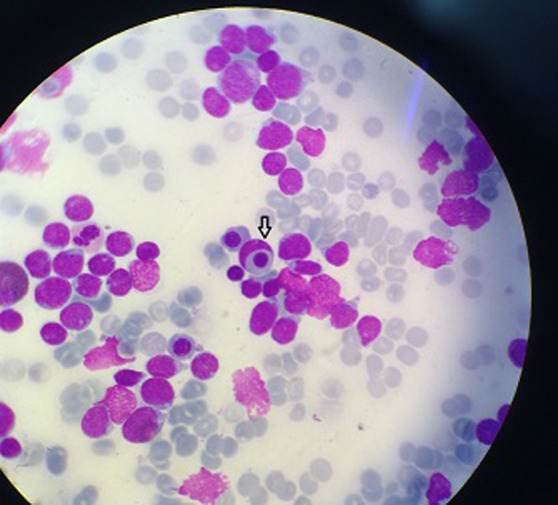
image montrant la phagocytose d’un érythroblaste par un lymphoblaste T (MGG× 100)

L´analyse des frottis médullaires colorés avec MGG a montré une moelle très riche montrant de très rares mégacaryocytes et envahie à 90% par des blastes. Par ailleurs il a été retrouvé sur les différents frottis médullaires examinés la présence d´une érythrophagocytose blastique (Figure 1, Figure 2, Figure 3). La réaction à la MPO a été négative. L´aspect cytologique a été en faveur d´une LAL selon la classification FAB mais avec une érythrophagocytose blastique. L´immunophénotypage du sang médullaire par cytométrie en flux, sur l´automate FC 500 BECKMAN COULTER a noté sur les 88% de cellules étudiées: **des marqueurs d´immaturité et d´activation** (CD34 81%, HLA-DR 0%) et **des marqueurs de la lignée T** (CD3 intracytoplasmique 93%, CD3 75%, CD5 89%, CD7 94%, CD2 97%). **Les marqueurs myéloïdes** (myélopéroxydase intracytoplsamique, CD117, CD33, CD13, CD11C, CD14, CD36) et **lymphoïdes B** (CD79a intracytoplasmique, CD19, CD22) ont été négatifs. Au total, ces aspects sont compatibles avec une leucémie lymphoblastique T (LAL-T). L´analyse cytogénétique a été réalisée mais elle n´a montré aucune anomalie. La patiente a été traitée selon le protocole GRAALL (Group for Research on Adult Acute Lymphoblastic Leukemia), mais devant la présence de plusieurs facteurs de mauvais pronostiques comme : l’hyperleucocytose initiale, l’atteinte du SNC, la corticorésistance et la surexpression du gène mdr1. Devant la présence de plusieurs facteurs de mauvais pronostiques: l´hyperleucocytose initiale, la cortico-résistance. La patiente a été adressée à l’Hôpital d’Instruction des Armées Percy (HIA Percy) pour une allogreffe géno-identique avec son frère HLA compatible.

## Discussion

L'érythrophagocytose par des blastes leucémiques est un phénomène rare. Elle forme une catégorie diagnostique, pronostique et thérapeutique distincte. Ces images sont retrouvées surtout dans la leucémie monoblastique aiguë (LAM5) et dans la leucémie myélomonocytaire aiguë (LAM4) de novo ou celles liées à la chimiothérapie (plus de 50% des cas). La présence de la t (8; 16) dans les leucémies myéloïdes est quasi constante. Elle est associée à un mauvais pronostic avec une médiane de survie de 4.7 mois dans les LAM secondaires. Dans ce cas les blastes ont une morphologie monocytoïde. Un examen méticuleux de la moelle osseuse peut indiquer la présence de cette entité de pronostic péjoratif et aider les cliniciens à prendre une décision clinique bien informée. En effet, la t (8;16) (p11;p13) résulte de la fusion entre le gène MYST3 (ou MOZ ou KAT6A) localisé en 8p11 et le gène CREB binding protein (CREBBP) localisé en 16p13 [1, 3, 4].

De rares cas d'érythrophagocytose par blastes myéloïdes associés à d'autres anomalies cytogénétiques ont été décrits dans la littérature. Il s´agit principalement de: LAM avec t (10; 17) (p13; p12); LAM avec t (16; 21) et fusion des gènes (TLS/FUS-ERG); LAM 0 avec t (9; 22).

En ce qui concerne les hémopathies lymphoïdes, seulement deux cas de leucémies lymphoblastiques B qu´ont été signalés jusqu' à présent: un cas avec t (12; 21) et fusion des gènes ETV6- RUNX1 et un autre cas avec comme anomalie cytogénétique la délétion du bras long du chromosome 20 (20q-) [5, 6]. Notre patiente a présenté une LAL T avec érythrophagocytose blastique et sans anomalie cytogénétique.

Le mécanisme de la phagocytose par les blastes leucémiques est inconnu. D’une façon générale, la phagocytose est un processus qui conduit à l'ingestion d’une particule par une cellule. Ceci implique une liaison via les récepteurs du complément CR1 (liaison C3b) et CR3 aux récepteurs Fc des IgG, et aux récepteurs pour fimbriae (gp 150). Bien qu'aucun de ces récepteurs n'ait été signalé sur les myéloblastes, certains chercheurs ont signalé la présence de récepteurs Fc dans les lymphoblastes [2]. L´implication de certaines cytokines (TNF et IL2) a également été postulé. Ce phénomène est très rare et n'est pas associé à des anomalies cytogénétiques ou moléculaires spécifiques [6]. En résumé, l'érythrophagocytose par les cellules néoplasiques dans la leucémie aiguë a été le plus souvent associée aux blastes myéloïdes avec t (8; 16) et réarrangements C-MOZ. L’érythrophagocytose par des blastes lymphoïdes reste très exceptionnelle d’autant plus avec une anomalie cytogénétique. Il existe une interaction entre : les différentes voies leucémogènes impliquées ; le mécanisme hypothétique de l'érythrophagocytose ; et les anomalies cytogénétiques trouvées. Cette interaction devrait être examinée et discutée de manière concise, afin de trouver à l'avenir une place pour cette entité exceptionnelle dans la classification de l'OMS [7].

## Conclusion

La découverte d´érythrophagocytose blastique, dans notre cas qui est une LALT, semblerait être le premier article. D’où la nécessité de préciser l´impact de ces images sur le plan diagnostique, pronostique et thérapeutique ainsi que l´étude des anomalies génétiques associées comme dans le cas des LAM [1, 8].
